# Serum Levels of *Candida albicans* 65-kDa Mannoprotein (CaMp65p) Correlate with Liver Disease in Patients with Alcohol Use Disorder

**DOI:** 10.3390/microorganisms13112458

**Published:** 2025-10-28

**Authors:** Julia T. Schnabl, Silvia Sandini, Peter Stärkel, Phillipp Hartmann

**Affiliations:** 1 Bishop’s School, La Jolla, CA 92037, USA; 2 Department of Pediatrics, University of California San Diego, La Jolla, CA 92093, USA; 3 Division of Gastroenterology, Hepatology & Nutrition, Rady Children’s Hospital San Diego, San Diego, CA 92123, USA; 4 National Centre for Drug Research and Evaluation, Istituto Superiore di Sanità, 00161 Rome, Italy; silvia.sandini@iss.it; 5 Department of Hepato-Gastroenterology, St. Luc University Hospital, Université Catholique de Louvain, 1200 Brussels, Belgium; peter.starkel@saintluc.uclouvain.be

**Keywords:** mycobiome, microbiota, alcoholic liver disease, MP65

## Abstract

Alcohol-associated liver disease is a global health burden with high morbidity and mortality, and the fungal microbiome is important for its progression. In particular, *Candida albicans* and *C. albicans*-reactive T helper 17 (Th17) cells contribute to alcohol-associated liver disease. Specific *C. albicans* antigens that activate Th17 cells during this disease are unknown. The *C. albicans* 65 kDa mannoprotein (CaMp65p) is one of the most abundant and immunodominant proteins of *C. albicans*, and is capable of eliciting robust T cell and interleukin (IL)-17A responses. The aim of this study was to measure levels of CaMp65p in serum of patients with alcohol use disorder and liver disease. Serum CaMp65p levels were measured in the serum of 60 patients with alcohol use disorder using an indirect competitive Enzyme-Linked Immunoabsorbent Assay (ELISA). Serum CaMp65p levels were correlated with liver disease severity. Serum CaMp65p levels positively correlated with several clinical and biochemical markers of liver injury and disease within the patient group with alcohol use disorder, including serum aspartate aminotransferase (AST; *R* = 0.33, *p* = 0.0092), alanine aminotransferase (ALT; *R* = 0.27, *p* = 0.037), gamma-glutamyl transferase (GGT; *R* = 0.35, *p* = 0.0055) and alkaline phosphatase (*R* = 0.36, *p* = 0.0052), and with the circulating M65 fragment of cytokeratin 18 (CK18-M65; *R* = 0.51, *p* = 0.0012), a marker of hepatocyte death. In addition, patients with alcohol use disorder in the upper quartile had significantly higher liver stiffness (*p* = 0.0022). Serum CaMp65p was significantly higher in patients with fibrosis stage F2–F4 as compared with patients with no or minimal fibrosis F0–F1 (*p* = 0.0082). The area under the curve (AUC) for detecting F2–F4 fibrosis was 0.70. Elevated serum CaMp65p levels are associated not only with more severe hepatic injury, but also with liver fibrosis in patients with alcohol use disorder. Therefore, CaMp65p may serve as a non-invasive biomarker for fibrosis assessment in patients with alcohol use disorder.

## 1. Introduction

Alcohol-associated liver disease is a prevalent chronic liver disease and a leading indication for liver transplantation in the United States [1]. Almost 50% of liver disease deaths were estimated to be related to alcohol consumption [2]. Patients with alcohol use disorder typically develop hepatic steatosis, which is characterized by accumulation of lipids and especially triglycerides in hepatocytes. Steatosis may develop liver inflammation, hepatocyte damage and ballooning, which is commonly called steatohepatitis [3]. Alcohol-associated steatohepatitis may progress to fibrosis and cirrhosis with continued alcohol consumption [4]. Patients with cirrhosis have an increased risk of developing hepatocellular carcinoma [4]. Women develop alcohol-associated liver disease after lower levels of alcohol exposure and often experience more severe disease progression compared with men [5]. However, the prevalence of alcohol use disorder and liver disease is higher in men [5]. There is currently no FDA-approved drug available for alcohol-associated liver disease, and the goal of therapy is alcohol abstinence.

Metabolic dysfunction-associated steatotic liver disease (MASLD) is another major etiology of liver disease globally [6]. A recent systematic review and meta-analysis reported that the prevalence of MASL is significantly higher in men than in women [7]. Estrogens are considered protective against MASLD. Therefore, postmenopausal women have a higher incidence of MASLD than age-matched men [8]. It has been recently recognized that alcohol intake and metabolic risk factors frequently coexist in patients with liver disease, which led to the concept of MetALD, a condition in which alcohol and overnutrition synergize to exacerbate steatosis, inflammation, and fibrosis [9]. Even moderate alcohol consumption leads to disease progression in MASLD [10], emphasizing the need for biomarkers that can help stratify risk and monitor disease activity in this population.

Changes in the composition and function of the gut microbiota are common in patients with alcohol-associated liver disease [11]. While many studies are focusing on the bacterial microbiome, less attention has been paid to the fungal microbiome (mycobiome). Fungi are a much smaller fraction of the gut microbiota, but they produce immunogenic cell wall components and proteins that can stimulate innate and adaptive immune responses [12]. Moreover, the mycobiome has been found to be involved in almost any hepatobiliary disease [12]. Various fungi were identified to exacerbate liver disease, including *Malassezia restricta* [13], *Meyerozyma guilliermondii* [14], and *Aspergillus flavus* [15], whereas other fungi, such as *Saccharomyces boulardii*, improve multiple forms of experimental liver disease in rodents [16,17,18,19]. One of the best studied fungal species is *Candida albicans* (*C. albicans*). It has been linked to more severe forms of MASLD [20]. However, *C. albicans* appears to play an even more central role in alcohol-associated liver disease than in MASLD [21]. Patients with alcohol use disorder and alcohol-associated hepatitis have lower fungal diversity and an increased relative abundance of *Candida* spp. in the feces. Clinical biomarkers of fungal involvement are still scarce in patients with alcohol-associated liver disease. However, higher serum anti-*Saccharomyces cerevisiae* antibodies (ASCA) as a systemic immune response to fungal products or fungi were associated with increased mortality in patients with alcohol-associated hepatitis [22] and cirrhosis [23]. Using fecal cultures, the absolute abundance of fungi and *C. albicans* was increased in patients with alcohol use disorder and, in particular, in patients with alcohol-associated hepatitis [24]. Of note, two weeks of alcohol abstinence in patients with alcohol use disease improved markers of liver disease, and decreased the fecal relative abundance of *C. albicans* and specific anti-*C. albicans* immunoglobulin G levels [25]. *C. albicans* contributes to ethanol-induced liver disease via different mechanisms in preclinical models. *C. albicans* produces and secretes the toxic peptide candidalysin, which can directly induce hepatocyte death [24]. Furthermore, *C. albicans*-specific T helper 17 (Th17) cells are increased in the circulation and present in the liver of patients with alcohol-associated liver disease. *C. albicans*-reactive Th17 cells migrate from the intestine to the liver, where they activate upon exposure to *C. albicans* antigens, secrete interleukin 17 (Il17) and contribute to ethanol-induced liver disease in mice [26]. However, the specific *C. albicans* antigens that activate Th17 cells and promote the alcohol-associated liver disease are currently unknown. Identifying reliable fungal antigens could therefore provide new diagnostic tools and mechanistic insights.

*C. albicans* is a major opportunistic fungal pathogen, and mannoproteins play important roles in fungal physiology and host–pathogen interactions [27]. The CaMp65p antigen, a 65 kDa mannoprotein encoded by the CaMp65p gene, is one of the most abundant, immunodominant and virulence-related proteins on the *C. albicans* cell surface [28,29]. It is involved in cell wall remodeling, morphogenesis, pathogenesis, adhesion to host epithelial tissues, and biofilm formation [30,31]. CaMp65p is also a potent immunogen, eliciting robust T cell and IL-17A responses [32,33,34]. Antibodies against CaMp65p have been detected in patients with systemic candidiasis [35,36].

In this study, we measured serum levels of CaMp65p in patients with alcohol use disorder and correlated them with liver disease severity. We found that serum CaMp65p levels positively correlated with several clinical and biochemical markers of liver injury and disease in patients with alcohol use disorder. In addition, patients with alcohol use disorder in the upper quartile had significantly higher liver stiffness.

## 2. Methods

### 2.1. Human Subjects

Our study included 60 patients with alcohol use disorder. As described [37], patients with alcohol use disorder were recruited from an alcohol treatment program in Brussels, Belgium. Patients with alcohol use disorder fulfilled the Diagnostic and Statistical Manual of Mental Disorders IV (DSM IV) criteria [38] of alcohol dependence with active alcohol consumption (self-reported > 60 g/day for more than one year). Patients with alcohol use disorder did not take antibiotics or immunosuppressive medications in the 2 months preceding enrollment. A complete medication and medical history were collected at admission. Other exclusion criteria were diabetes, inflammatory bowel diseases, liver diseases of any other etiology, and clinically significant cardiovascular, pulmonary, or renal comorbidities. Basic demographic data (such as age, gender, weight, and height) and self-reported daily alcohol consumption were collected, and patients underwent a physical examination. On the day of admission, FibroScan (Echosense, Paris, France) with controlled attenuation parameter (CAP) and liver stiffness in kPa was performed, and blood samples were collected.

### 2.2. Ethics Approval and Consent

As described previously [25], the study protocol conforms to the ethical guidelines of the 1975 Declaration of Helsinki and was approved by the institution’s human research and ethical committee (Université Catholique de Louvain, Brussels, Belgium; B403201422657). Written informed consent was obtained from all patients.

### 2.3. Serum Biomarkers

Standard biochemical serum studies, including measurement of aspartate aminotransferases (AST), alanine aminotransferase (ALT), bilirubin, gamma-glutamyltransferase (GGT), and alkaline phosphatase (AP), were performed at the clinical laboratory associated with St. Luc University Hospital, Brussels, Belgium. Additionally, serum intact cytokeratin 18 (CK18-M65) was used to assess liver cell damage (CK18-M65 ELISA kit; TECOmedical AG, Sissach, Switzerland) [39].

### 2.4. Indirect Competitive ELISA for Serum CaMp65p Antigen Detection and Quantification

To quantify serum *C. albicans* Mp65 antigen in humans, we designed a custom-made indirect competitive ELISA. Briefly, we coated a polystyrene 96-well microtiter plate with purified, recombinant CaMp65p (rCaMp65p, synthesized by a genetically modified *Escherichia coli* strain (M15-pUHA1-pRLV130) [29]; 10 µg/mL protein in phosphate-buffered saline (PBS), pH 7.4, 50 µL/well) overnight at +4 °C before washing with PBS containing 0.05% *v*/*v* Tween^®^-20 and blocking with PBS containing 1% *w*/*v* bovine serum albumin (BSA) for 2 h at 37 °C. In another polystyrene 96-well microtiter plate, we incubated human sera (the test sample: antigen) with a murine anti-CaMp65p monoclonal 4c8 (mAb 4c8) IgG_1_ antibody [28,29,40] in a 1:1 dilution for 1 h at 37 °C. Then, we added 50 µL of these incubated sera to each well of the coated 96-well microtiter plate and incubated for 1 h at 37 °C. After washing, we added a reporter anti-mouse IgG (heavy chain) secondary antibody, labeled with horseradish peroxidase (HRP) (ThermoFisher Scientific, Waltham, MA, USA, # 31430) and incubated for 1 h, before washing. Finally, we added the substrate 3,3′,5,5′-tetramethylbenzidine (TMB) (ThermoFisher Scientific, # 00-4201-56) and stop solution (sulfuric acid), and read the optical density at 450 nm to determine the relative CaMp65 serum levels. Pooled undiluted and diluted human serum samples were used as standards. Negative controls included PBS samples. Specificity of the primary mAb 4c8 and hence the ELISA was confirmed by varying the concentrations of the individual components, including the coating rCaMp65p and the mAb 4c8.

### 2.5. Statistics

The Mann–Whitney U test/Wilcoxon rank-sum test was used, and results are expressed as median and range, except when stated otherwise. Pearson correlation analysis of serum CaMp65p levels with disease parameters was performed. A *p* value less than 0.05 was considered statistically significant. Area under the curve (AUC), best threshold to maximize the Youden Index, sensitivity, specificity, accuracy, positive predictive value, and negative predictive value were calculated using the pROC library in R. Statistical analysis was performed using R statistical software, R version 2024.12.1 for Mac, the R Foundation for Statistical Computing.

## 3. Results

### 3.1. Study Population

The study population included 60 patients with alcohol use disorder and varying degrees of alcohol-associated liver disease (Table 1). There were predominantly male subjects with a mean age of 46.5 years and a body mass index within normal limits. As expected, patients with alcohol use disorder had elevated liver function tests, including aminotransferase (AST), alanine aminotransferase (ALT) and gamma-glutamyl transferase (GGT). The mean of alkaline phosphatase (AP), bilirubin, albumin, international normalized ratio (INR), creatinine and platelets was within normal limits. Patients with alcohol use disorder had varying degrees of liver disease: 53.3% had stage F0/1 fibrosis, 11.7% had F2 fibrosis, 15% had F3 fibrosis, and 20% had cirrhosis (Table 1).

### 3.2. CaMp65p Serum Levels Correlate with Markers of Liver Disease Severity in Patients with Alcohol Use Disorder

**Figure 1:**
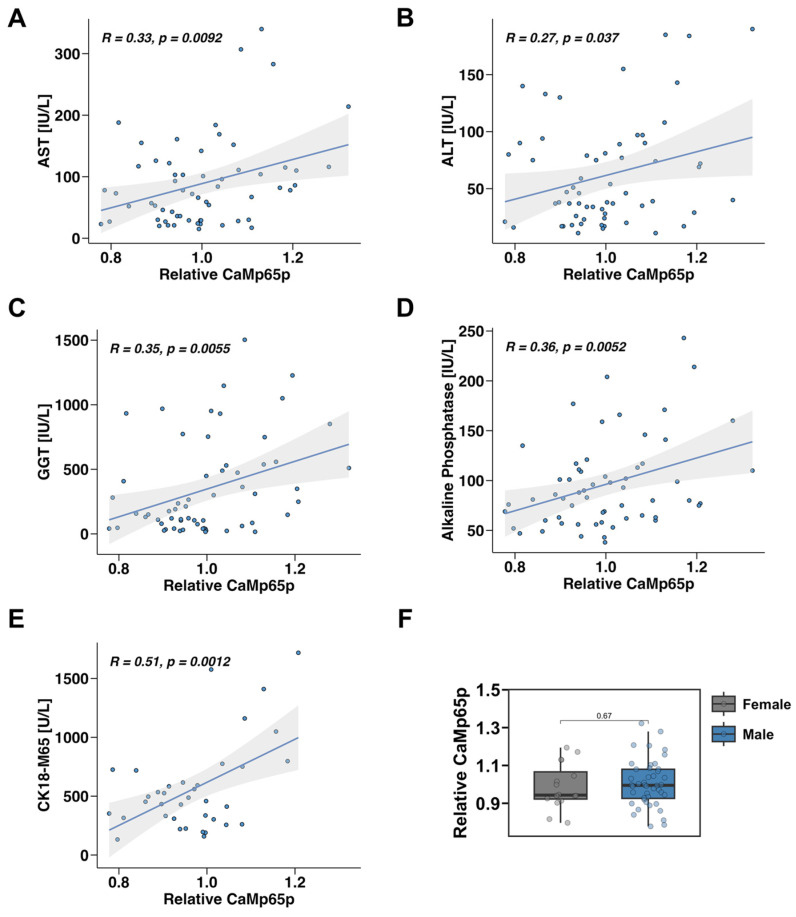
Correlation between serum CaMp65p and liver disease parameters in patients with alcohol use disorder. Pearson correlation analysis of serum CaMp65p levels with serum aspartate aminotransferase (AST) (**A**), alanine aminotransferase (ALT) (**B**), gamma-glutamyl transferase (GGT) (**C**), alkaline phosphatase (**D**), and of the M65 fragment of cytokeratin 18 (CK18-M65) (**E**). (**F**) Relative serum CaMp65p levels in male vs. female patients with alcohol use disorder.

Serum CaMp65p levels positively correlated with several clinical and biochemical markers of liver injury and disease. Specifically, higher CaMp65p levels were associated with increased serum AST (Figure 1A), ALT (Figure 1B), GGT (Figure 1C), and alkaline phosphatase (AP) (Figure 1D). The strongest correlation was found with the circulating M65 fragment of cytokeratin 18 (CK18-M65), a marker of hepatocyte death (Figure 1E). These findings indicate that elevated CaMp65p correlates with liver injury in patients with alcohol use disorder. There was no significant difference in relative serum CaMp65p levels in male versus female patients with alcohol use disorder (Figure 1F).

### 3.3. Higher CaMp65p Levels Stratify Patients with More Severe Alcohol-Associated Liver Disease

**Figure 2:**
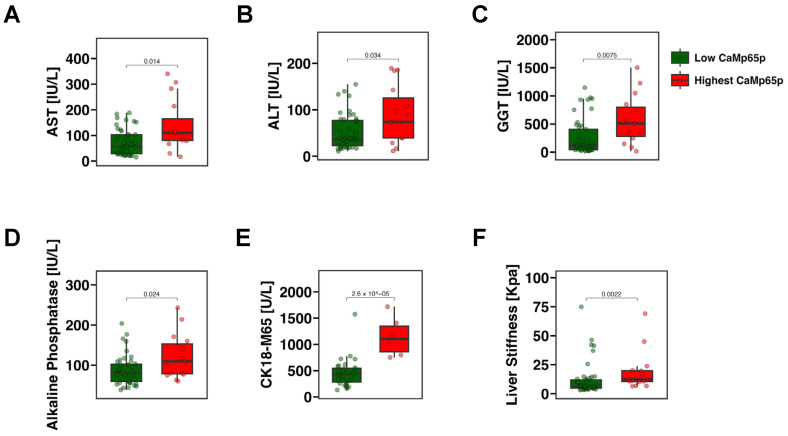
Markers of liver damage and fibrosis stratified by CaMp65p levels in patients with alcohol use disorder. Comparison between patients in the upper quartile vs. the lower three quartiles of CaMp65p serum levels. Serum aspartate aminotransferase (AST) (**A**), alanine aminotransferase (ALT) (**B**), gamma-glutamyl transferase (GGT) (**C**), alkaline phosphatase (**D**), the M65 fragment of cytokeratin 18 (CK18-M65) (**E**), and liver stiffness (**F**) in patients with alcohol use disorder stratified by CaMp65p quartile. Statistical comparison was performed using the Mann–Whitney U test.

To examine whether CaMp65p can stratify patients according to disease severity, patients with alcohol use disorder were divided into the upper quartile (top 25%) and the lower three quartiles (bottom 75%) based on relative serum CaMp65p levels. Individuals in the upper quartile exhibited significantly higher serum AST (Figure 2A), ALT (Figure 2B), GGT (Figure 2C), alkaline phosphatase (Figure 2D), and CK18-M65 levels (Figure 2E) compared with those in the lower three quartiles. In addition, patients with alcohol use disorder in the upper quartile had significantly higher liver stiffness as determined by FibroScan (Figure 2F), indicating that elevated CaMp65p is associated not only with more severe hepatic injury, but also liver fibrosis.

### 3.4. Serum CaMp65p Levels Are Associated with Liver Fibrosis Stage in Patients with Alcohol Use Disorder

**Figure 3:**
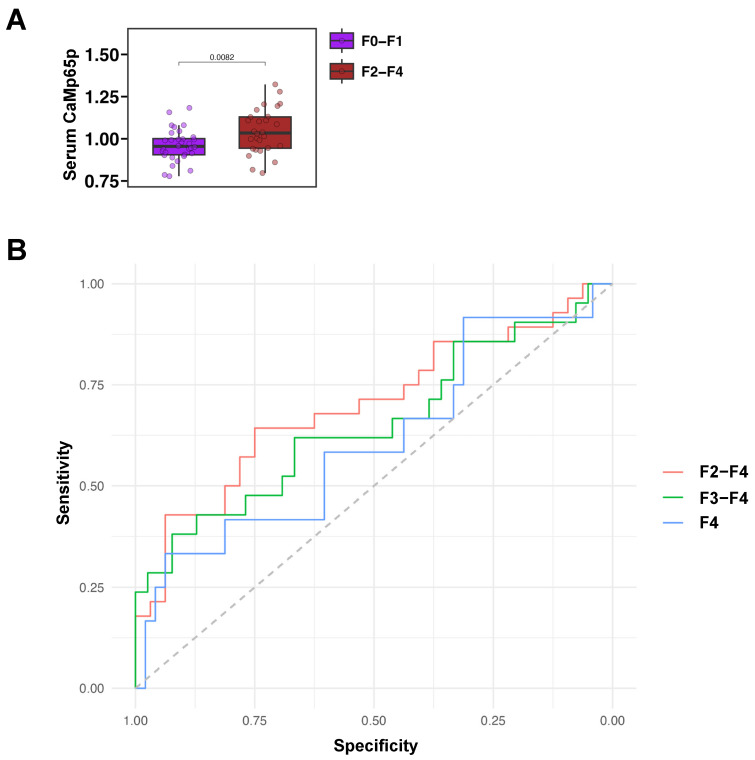
Serum CaMp65p levels by fibrosis stage and associated receiver operating characteristic curve analysis in patients with alcohol use disorder. (**A**) Serum CaMp65p levels in patients with higher fibrosis stages F2–F4 vs. F0–F1. Statistical comparison was performed using the Mann–Whitney U test. (**B**) Receiver operating characteristic curve (ROC) performance of serum CaMp65p levels in detecting fibrosis stage F2–F4, F3–F4, and F4.

Serum CaMp65p levels were next analyzed in relation to fibrosis stage. Serum CaMp65p was significantly higher in patients with fibrosis stage F2–F4 as compared with patients with no or minimal fibrosis F0–F1 (Figure 3A). A receiver operating characteristic curve (ROC) analysis demonstrated that CaMp65p has diagnostic value in identifying fibrosis stages. The area under the curve (AUC) for detecting F2–F4 fibrosis was 0.70, for F3–F4 fibrosis was 0.65, and for F4 fibrosis was 0.61 (Figure 3B, Table 2). Serum CaMp65p demonstrated best performance in predicting F2–F4 vs. F0–F1 fibrosis compared with predicting F3–F4 or F4, with a Youden Index of 0.39, a sensitivity of 0.64, specificity of 0.75, accuracy of 0.70, a positive predictive value (PPV) of 0.69, and negative predictive value (NPV) of 0.71 (Table 2).

## 4. Discussion

This study identifies serum CaMp65p, a major mannoprotein antigen of *C. albicans* [28,31], as a potential non-invasive biomarker of liver disease severity in patients with alcohol use disorder. The observed correlations between serum CaMp65p levels and AST, ALT and CK18-M65 indicate that CaMp65p is closely linked to liver injury. CaMp65p is involved in cell wall remodeling, morphogenesis, pathogenesis, adhesion to host epithelial tissues, and biofilm formation [30,31] but is, in particular, one of the most immunodominant and virulence-related proteins on the *C. albicans* cell surface [28,29], which would explain its contribution to liver disease. Furthermore, the stratification of patients with alcohol use disorder by CaMp65p levels revealed that individuals in the highest quartile had significantly greater biochemical evidence of liver damage. Notably, CaMp65p levels were significantly associated with fibrosis stage. This indicates that CaMp65p could serve as a marker to identify patients at risk for more severe disease. These findings go beyond previous studies, which showed increased fecal relative abundance of *C. albicans* in alcohol-associated liver disease [22,23]. They are in line with prior blood biomarker data on ASCA [22,23] and anti-*C. albicans* immunoglobulins G and M in alcohol-associated liver disease [25]. However, the new biomarker is even more specific than the reported serologies and is related to just a portion of a fungal pathobiont. While the sensitivity of CaMp65p in identifying fibrosis stages is modest, its relatively higher specificity suggests greater utility as a rule-in tool for detecting significant fibrosis. This may be particularly valuable in clinical settings where confirming the presence of fibrosis is critical for decision-making, such as prioritizing patients for further diagnostic workup or treatment. These findings are particularly relevant given the need for better non-invasive tools to identify advanced fibrosis in patients with alcohol use disorder. Future studies are warranted to define and validate an optimal threshold for CaMp65p that enhances its performance as a confirmatory marker in fibrosis assessment.

These results also support prior findings showing that fungal translocation and immune responses to fungal antigens contribute to liver disease in preclinical models. While candidalysin and *C. albicans*-reactive Th17 cells have been implicated in preclinical models [24,26], CaMp65p represents a novel, measurable, and immunologically relevant candidate in alcohol-associated liver disease. Its known ability to elicit T cell responses [32,33], as well as its role in fungal adhesion [30,31], may explain its relevance in disease pathogenesis. However, additional studies in experimental models of ethanol-induced liver disease using CaMp65p-specific interventions and immune responses are necessary to confirm this notion.

The association between CaMp65p and biochemical markers of liver injury indicates that systemic exposure to fungal antigens may directly lead to hepatocellular damage and inflammation in alcohol-associated liver disease. Elevated CaMp65p could reflect increased intestinal permeability and fungal translocation, which is known to accompany ethanol-induced gut barrier disruption. The correlation with CK18-M65, a marker of hepatocyte death, further supports the concept that fungal components exacerbate hepatic injury by stimulating immune pathways such as Th17 activation and cytokine release. Thus, CaMp65p may not only serve as a biomarker but also indicate a mechanistic link between fungal antigenemia and liver injury severity. Fibrosis might be the consequence of chronic liver injury.

From a translational perspective, fungal biomarkers might lead to novel therapeutic approaches. Antifungal strategies, prebiotics, probiotics, or barrier-protective agents could be tested in preclinical models of ethanol-induced liver disease with CaMp65p titers as readouts.

Limitations of this study include its single-center design and the cross-sectional nature of the data, which preclude conclusions about causality or progression over time. Furthermore, head-to-head comparisons with bacterial and host-derived biomarkers would clarify whether CaMp65p adds independent diagnostic value. Future studies should examine the predictive value of CaMp65p longitudinally.

## 5. Conclusions

CaMp65p represents a novel biomarker candidate for alcohol-associated liver disease. Beyond its diagnostic potential as a biomarker, our study shows the importance of the mycobiome in alcohol-associated liver disease. While bacterial translocation has long been recognized in alcohol-associated liver disease, fungal products may contribute equally to inflammation and fibrosis progression. Our study provides a rationale for integrating fungal biomarkers into clinical research pipelines.

Future directions include: (1) validation of CaMp65p in larger, multi-center cohorts; (2) longitudinal studies to determine its ability to predict fibrosis progression; (3) mechanistic experiments in animal models to link MP65 exposure with ethanol-induced hepatic immune activation, liver steatosis and fibrosis; and (4) exploration of therapeutic interventions that reduce fungal burden or increase barrier integrity. Collectively, these efforts could establish CaMp65p as both a clinical tool and a therapeutic target.

In addition, future research should explore whether serum CaMp65p levels can distinguish alcohol-associated liver disease from other etiologies, such as MASLD or MetALD, where fungal dysbiosis may also contribute to hepatic inflammation. Comparative profiling of fungal antigens across distinct liver disease populations could reveal shared or disease-specific mycobiome signatures. Integrating CaMp65p quantification with established noninvasive fibrosis markers, such as transient elastography or serum fibrosis panels, may improve diagnostic accuracy. Ultimately, a multi-omic approach combining bacterial, fungal, and host biomarkers could advance individualized risk assessment and therapeutic stratification in alcohol-associated and metabolic liver diseases.

This work provides the first clinical evidence that serum CaMp65p associates with severity of alcohol-associated liver disease and may serve as a useful non-invasive biomarker.

## Figures and Tables

**Table 1:** Demographics, laboratory and clinical parameters of patients with alcohol use disorder.

Variables	Alcohol Use Disorder (n = 60)
**Gender (% male)** **n = 60**	44 (73.3%)
**Age (years)** **n = 60**	46.5 ± 11.9
**BMI** **n = 60**	24.8 ± 3.9
**AST (IU/L)** **n = 60**	88.8 ± 71.4
**ALT (IU/L)** **n = 60**	61.6 ± 46.8
**GGT** **n = 60**	347.2 ± 367.9
**AP (IU/L)** **n = 59**	96.4 ± 44.9
**Bilirubin (mg/dL)** **n = 59**	0.7 ± 0.8
**Albumin (g/dL)** **n = 58**	45.5 ± 6
**INR** **n = 57**	1 ± 0.2
**Creatinine (mg/dL)** **n = 60**	0.7 ± 0.2
**Platelets** **n = 38**	226.7 ± 82.4
**CAP** **n = 60**	295.4 ± 58.5
**Stage of Fibrosis, n (%)**	
**F0/1**	32 (53.3%)
**F2**	7 (11.7%)
**F3**	9 (15%)
**F4**	12 (20%)

Data is expressed as mean and standard deviation for each continuous outcome. Values are presented as percentages in parentheses for categorical variables. Percentages are calculated based on the actual number of patients in each group, where the respective data was available. The number of subjects for which the respective data was available is indicated in the first column. AP, alkaline phosphatase; ALT, alanine aminotransferase; AST, aspartate aminotransferase; BMI, body mass index; CAP, controlled attenuation parameter; GGT, gamma-glutamyl transferase; INR, international normalized ratio.

**Table 2:** Area under curve for serum CaMp65p levels in different fibrosis categories.

Marker	AUC	Youden	Threshold	Sens.	Spec.	Accuracy	PPV	NPV
CaMp65p for F2–F4	**0.70**	0.39	1.00	0.64	0.75	0.70	0.69	0.71
CaMp65p for F3–F4	**0.65**	0.30	1.11	0.38	0.92	0.73	0.73	0.73
CaMp65p for F4	**0.61**	0.27	1.16	0.33	0.94	0.82	0.57	0.85

The best threshold was determined to maximize the Youden index (= sensitivity + specificity − 1) for each fibrosis stage. AUC, area under curve; NPV, negative predictive value; PPV, positive predictive value; Sens., sensitivity; Spec., specificity.

## Data Availability

The original contributions presented in this study are included in the article. Further inquiries can be directed to the corresponding author.

## Abbreviations

ALT, alanine aminotransferase; AST, aspartate aminotransferase; AUC, area under curve; AUD, alcohol use disorder; BMI, body mass index; CK18-M65, M65 fragment of cytokeratin 18; Ctrl, controls; GGT, gamma-glutamyl transferase; INR, international normalized ratio; MASLD, Metabolic dysfunction-associated steatotic liver disease; ROC, receiver operating characteristic curve.
